# Assessing COVID-19 Health Information on Google Using the Quality Evaluation Scoring Tool (QUEST): Cross-sectional and Readability Analysis

**DOI:** 10.2196/32443

**Published:** 2022-02-11

**Authors:** Vismaya S Bachu, Heba Mahjoub, Albert E Holler, Tudor Crihalmeanu, Dheevena M Bachu, Varun Ayyaswami, Pearman D Parker, Arpan V Prabhu

**Affiliations:** 1 School of Medicine Johns Hopkins University Baltimore, MD United States; 2 School of Medicine West Virginia University Morgantown, WV United States; 3 Department of Neuroscience University of Colorado Boulder, CO United States; 4 Department of Internal Medicine University of Massachusetts Medical School Worcester, MA United States; 5 College of Nursing University of Arkansas for Medical Sciences Little Rock, AR United States; 6 Department of Radiation Oncology University of Arkansas for Medical Sciences Winthrop P Rockefeller Cancer Institute Little Rock, AR United States

**Keywords:** COVID-19, COVID-19 pandemic, health literacy, readability, QUEST, online health information, cross-sectional, trend, internet, spread, symptom, quality, United States

## Abstract

**Background:**

The COVID-19 pandemic spurred an increase in online information regarding disease spread and symptomatology.

**Objective:**

Our purpose is to systematically assess the quality and readability of articles resulting from frequently Google-searched COVID-19 terms in the United States.

**Methods:**

We used Google Trends to determine the 25 most commonly searched health-related phrases between February 29 and April 30, 2020. The first 30 search results for each term were collected, and articles were analyzed using the Quality Evaluation Scoring Tool (QUEST). Three raters scored each article in authorship, attribution, conflict of interest, currency, complementarity, and tone. A readability analysis was conducted.

**Results:**

Exactly 709 articles were screened, and 195 fulfilled inclusion criteria. The mean article score was 18.4 (SD 2.6) of 28, with 7% (14/189) scoring in the top quartile. National news outlets published the largest share (70/189, 36%) of articles. Peer-reviewed journals attained the highest average QUEST score compared to national/regional news outlets, national/state government sites, and global health organizations (all *P*<.05). The average reading level was 11.7 (SD 1.9, range 5.4-16.9). Only 3 (1.6%) articles were written at the recommended sixth grade level.

**Conclusions:**

COVID-19–related articles are vastly varied in their attributes and levels of bias, and would benefit from revisions for increased readability.

## Introduction

Since the onset of the COVID-19 pandemic, new information is released daily, if not hourly, regarding disease spread, symptomatology, and health and economic consequences. In some cases, news has been rapidly spread only to be contradicted days later. For example, at the beginning of the pandemic, hydroxychloroquine was regularly discussed in lay news and scientific journals alike. Some articles touted a 90% chance of benefit to patients with COVID-19 [1], while peer-reviewed journals soon thereafter released a lack of clinical improvement with use of the drug [2,3]. Given varying accuracy levels of innumerable sources, there is a clear need for standardized quality control of online health information especially in light of current vaccination and other public health campaigns [4].

There was a disjointed public health response, partly due to contradicting information. For example, we now know that universal masking is of the utmost importance in preventing disease transmission, but earlier in the pandemic, it was only recommended for health care professionals [1,3]. These conflicting messages may have left many consumers confused, frustrated, and unsure of what broadcast news channels and online health information to trust. The burden of sorting through the flood of information fell on the consumer and, in many instances, left the consumer feeling paralyzed with information overload and overconcern from frequent use of social media [5]. Furthermore, an analysis of online health information prior to February 6, 2020, showed low quality information relative to several different quality scoring systems, including HONcode, the JAMA benchmark, and the DISCERN instrument [6]. With the prevalence of low-quality information and sudden influx of new conflicting information and associated overwhelming emotion, we felt compelled to analyze the information being consumed by the public.

Google Trends (GT) was used to identify popular COVID-19 search terms and produce a list of related online health articles, after which the Quality Evaluation Scoring Tool (QUEST) was applied to assess validity. QUEST is a verified metric created to assess online health information, or any information available online that patients may read to learn more about their health, in a quantifiable way. It consists of seven questions that numerically measure quality of authorship, attribution, conflict of interest, currency, complementarity, and tone [7]. According to QUEST, a high-quality article is deemed trustworthy and credible, and displays an appropriate level of tone for the reader. We opted for this tool, as opposed to another scale such as DISCERN, because it provides clear guidelines on scoring, with example statements clarifying which articles should receive a score of 0 to 3. Furthermore, the scores are weighted, emphasizing the importance of attribution, conflict of interest, and tone in assessing quality. Though there are many unique tools to analyze online health information, we valued QUEST for its unambiguous scoring and similarity to the US National Library of Medicine’s “Medline Plus Guide” in individually judging legitimacy of online health material [8].

In addition to systematically assessing the quality of articles using QUEST, we sought to evaluate the readability of articles resulting from the most frequently Google-searched health-related COVID-19 terms in the United States. Because it is additionally important to recognize the varying degrees of literacy within the public, a readability analysis was performed on each article to compare against the recommended sixth grade reading level for patient health communication materials [9]. Although the production of accurate health information for patients to consume is important, it is equally important for the information to be presented in an understandable manner [10]. We hypothesized that the reading levels of popularly searched health phrases would be too difficult for the average American to understand and that the public was consuming low quality online information regarding COVID-19.

## Methods

### Article Selection

**Figure 1:**
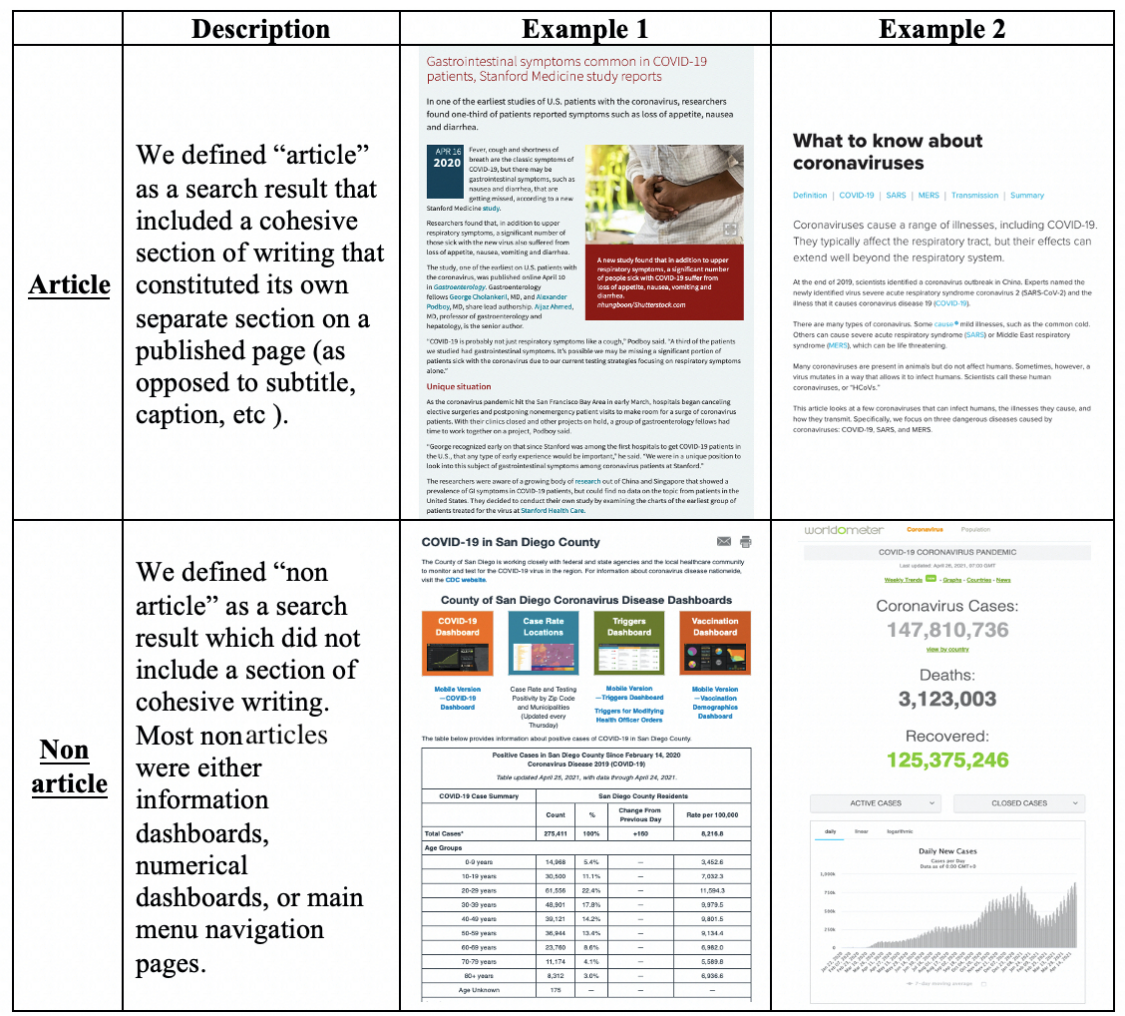
Description and examples of basic inclusion (article) vs exclusion (nonarticle) criteria. Article, Example 1: “Gastrointestinal Symptoms Common in COVID-19 Patients, Stanford Medicine Study Reports.” Article, Example 2: “What to Know about Coronaviruses.” Nonarticle, Example 1: “COVID-19 in San Diego County.” Nonarticle, Example 2: “Covid-19 Coronavirus Pandemic."

Institutional review board approval was not required for this study since all information was freely available online. For the purposes of this study, we defined an “article” as being any piece of published writing excluding personal blogs, editorials, and commentaries (Figure 1).

GT has been widely used to capture the most popular queries searched by the public, providing important information regarding emerging patterns. Prior studies have supported the use of GT to monitor COVID-19 incidence and public attention, especially within countries lacking proper diagnostic tools [11,12]. To prevent location bias, online articles were collected using a location-disabled search on Google.com/ncr on April 30, 2020. Using GT, we used the “Explore” option and applied the parameters “United States,” “Custom date range 2/29/20 to 4/30/20,” “Health,” and “Web search.” A start date of February 29, 2020, was chosen because this was associated with the first Centers for Disease Control and Prevention–reported death from COVID-19 in the United States [13] and marks an increase in Google searches for the word “coronavirus.” From here, we sorted the search queries by “Top” and then collected the top five search queries that had an increased level of Google search frequency (Figure 2.1). These were “coronavirus,” “corona,” “corona virus,” “symptoms,”and “coronavirus update.” We then used each of these terms and searched them on GT using the aforementioned methodology. Along with the original five terms, we collected the top five related search queries (including the initially searched word) and excluded any repeat search queries. This resulted in 25 unique health-related search phrases (Figure 2.2). Of note, multi-word keywords were searched using quotations marks, and a comparative analysis of search volume without quotes was not performed.

Next, between April 30 and May 2, 2020, we searched each keyword phrase and collected all articles (writing that includes more than 100 words) from the first 3 pages of the Google search; this resulted in approximately 10 articles per page (Figure 3). Prior research has shown that internet users tend not to view past these first 3 pages on Google [14]. When queries yielded articles that overlapped, we excluded the repeated articles from analysis. For each article, we collected the Google page number, order on the page, article link, website name, category of website, article title, author, date of publication, and number of references.

**Figure 2:**
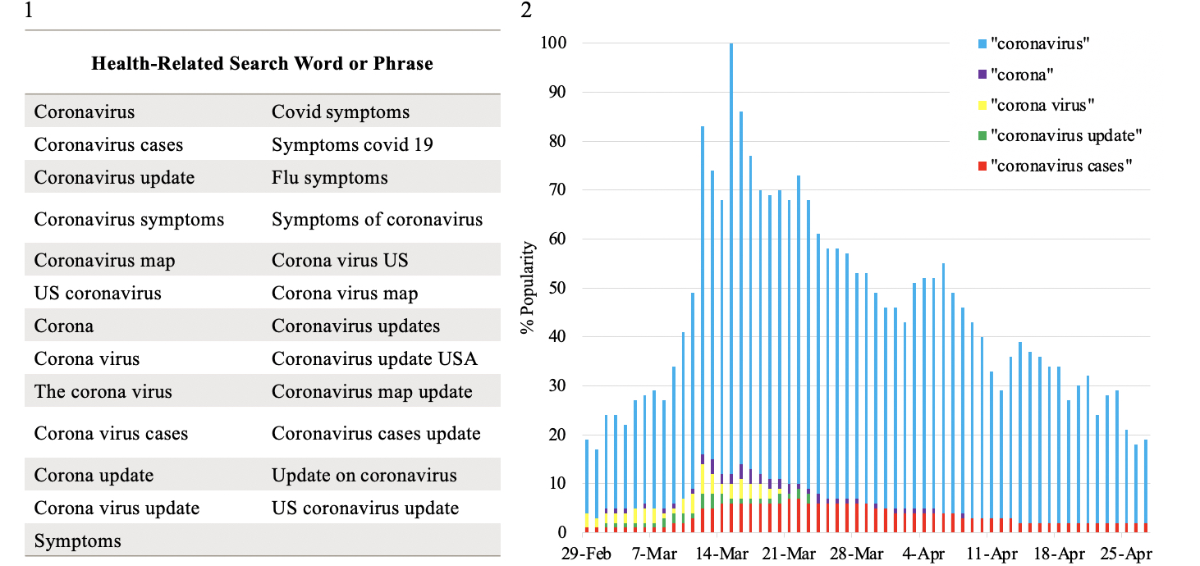
(1) Google search words/phrases used to collect most viewed articles. (2) Increase in Google search popularity of the five most commonly searched health-related phrases in the United States between February 29 to April 30, 2020.

**Figure 3:**
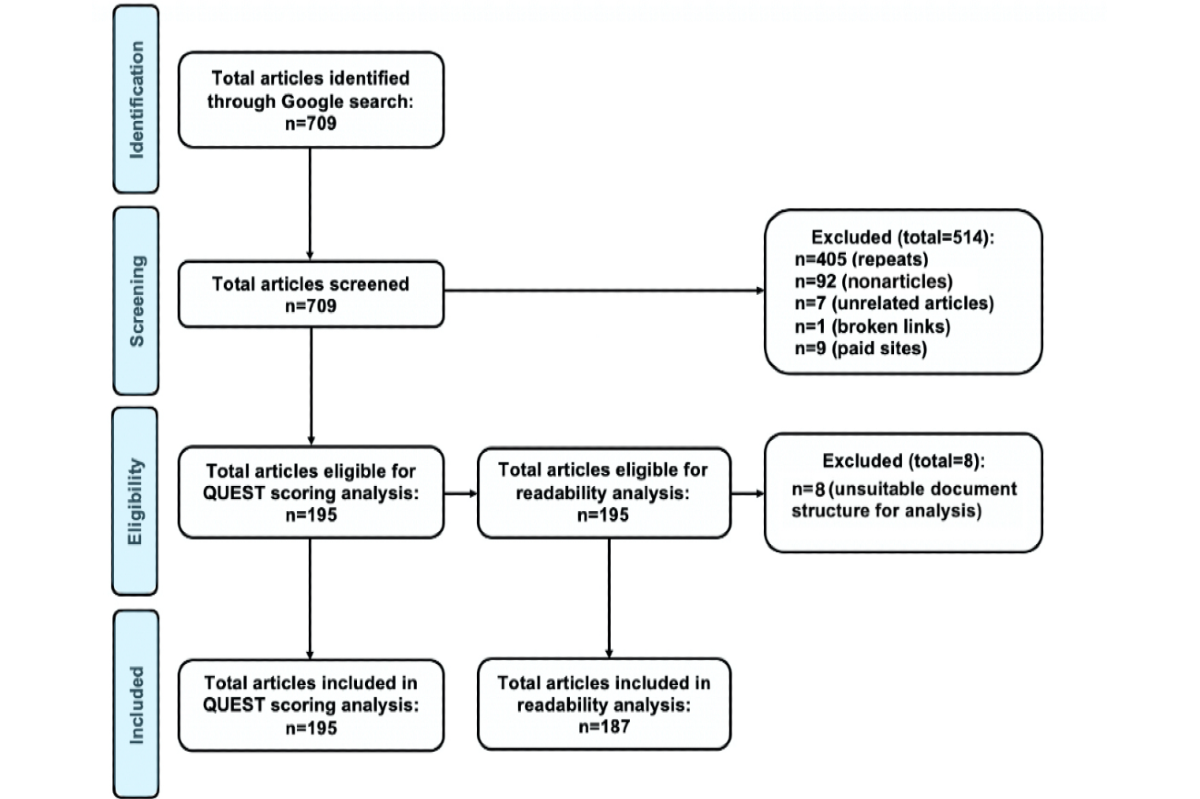
CONSORT (Consolidated Standards of Reporting Trials) diagram depicting article flow and eventual sample size. QUEST: Quality Evaluation Scoring Tool.

### QUEST Scoring

Three separate authors scored all articles individually using each of the 7 QUEST questions and associated point values (Textbox 1). Each article’s individual sections were then combined into a score between 0 and 28, where 28 was the highest quality article possible. The final score for each article was an average of the three independent scorers’ analyses. Interrater consensus was determined using Fleiss’s kappa metric.

**The Quality Evaluation Scoring Tool scoring rubric.**

**Authorship (score x1)**

- 0—No indication of authorship or username
- 1—All other indications of authorship
- 2—Author’s name and qualification clearly stated

**Attribution (score x3)**

- 0—No sources
- 1—Mention of expert source, research findings (though with insufficient information to identify the specific studies), links to various sites, advocacy body, or other
- 2—Reference to at least one identifiable scientific study, regardless of format (eg, information in text or reference list)
- 3—Reference to mainly identifiable scientific studies, regardless of format (in >50% of claims)
  For all articles scoring 2 or 3 on attribution: type of study (score x 1): 0—in vitro, animal models, or editorials; 1—all observational work; 2—meta-analyses, randomized controlled trials, clinical studies

**Conflict of interest (score x3)**

- 0—Endorsement or promotion of intervention designed to prevent or treat condition (eg, supplements, brain training games, or foods) within the article
- 1—Endorsement or promotion of educational products and services (eg, books or care home services)
- 2—Unbiased information

**Currency (score x1)**

- 0—No date present
- 1—Article is dated but 5 years or older
- 2—Article is dated within the last 5 years

**Complementarity (score x1)**

- 0—No support of the patient-physician relationship
- 1—Support of the patient-physician relationship

**Tone (includes title; score x3)**

- 0—Fully supported (authors fully and unequivocally support the claims, strong vocabulary; eg, “cure,” “guarantee,” and “easy”; mostly use of nonconditional verb tenses [“can,” “will”], no discussion of limitations)
- 1—Mainly supported (authors mainly support their claims but with more cautious vocabulary; eg, “can reduce your risk” or “may help prevent”; no discussion of limitations
- 2—Balanced/cautious support (authors’ claims are balanced by caution, includes statements limitations or contrasting findings)

### Statistical Analysis

Excel (Microsoft Corporation) was used to conduct the statistical analysis and generate figures for this study. In addition to determining general descriptive metrics (mean, median, etc), we coined the metric search order product to examine if there was any statistical difference in quality between articles toward the beginning and end of the results. Equal to the results page number on Google multiplied by the order of article on that page, the search order product encompasses the article’s hierarchy in search results. For example, if an article was second on the third page of the results on Google, its search order product would be 6. A low search order product indicates an earlier appearing article once its associated phrase is searched (meaning increased public exposure), while a high search order product is characteristic of a later-appearing article (decreased public exposure). Comparative *t* tests and Pearson correlation analyses were conducted to stratify scores by variables. The Benjamini-Hochberg false detection rate correction for multiplicity was applied to appropriately adjust P values.

### Readability Analysis

Readability analysis was performed using Readability Studio Professional Edition Version 2015 (Oleander Software, Ltd), applying nine validated formulas to quantify article readability: Coleman-Liau Index [15], Flesch-Kincaid Grade Level [16], FORCAST formula [17], Fry graph [18], Gunning Fog Index [19], New Dale-Chall [20], New Fog Count [16], Raygor Reading Estimate [21], and SMOG (Simple Measure of Gobbledygook) [22]. Nine different readability scales were used to minimize the bias that comes with using only one scale. We subsequently calculated the reading level for each article by averaging estimates derived from all nine scales. These were then compared to the American Medical Association–recommended reading level of sixth grade for health education materials [9]. A 10th readability formula, the Flesch Reading Ease (FRE) [23], was applied separately as it calculates reading level on a different scale. FRE scores of 0 to 30 indicate very difficult, 30 to 50 difficult, 50 to 60 fairly difficult, 60 to 70 standard, 70 to 80 fairly easy, 80 to 90 easy, and 90 to 100 very easy.

## Results

### QUEST Analysis

A total of 709 Google results listings were initially examined. After excluding repeated articles (n=405), nonarticles (n=92), unrelated articles (n=7), broken links (n=1), and paid sites (n=9) from the analysis, 195 individual articles were scored using QUEST (Figure 3).

The mean article score was 18.4 (SD 2.6) of 28, with only 7% (14/189) of articles in the top score quartile and 89% (173/189) of articles in the top half of scores. National news outlets published the largest share (70/189, 36%) of the analyzed articles, followed by private health-focused entities (45/189, 23%) and regional news outlets (29/189, 15%; Table 1).

**Figure 4:**
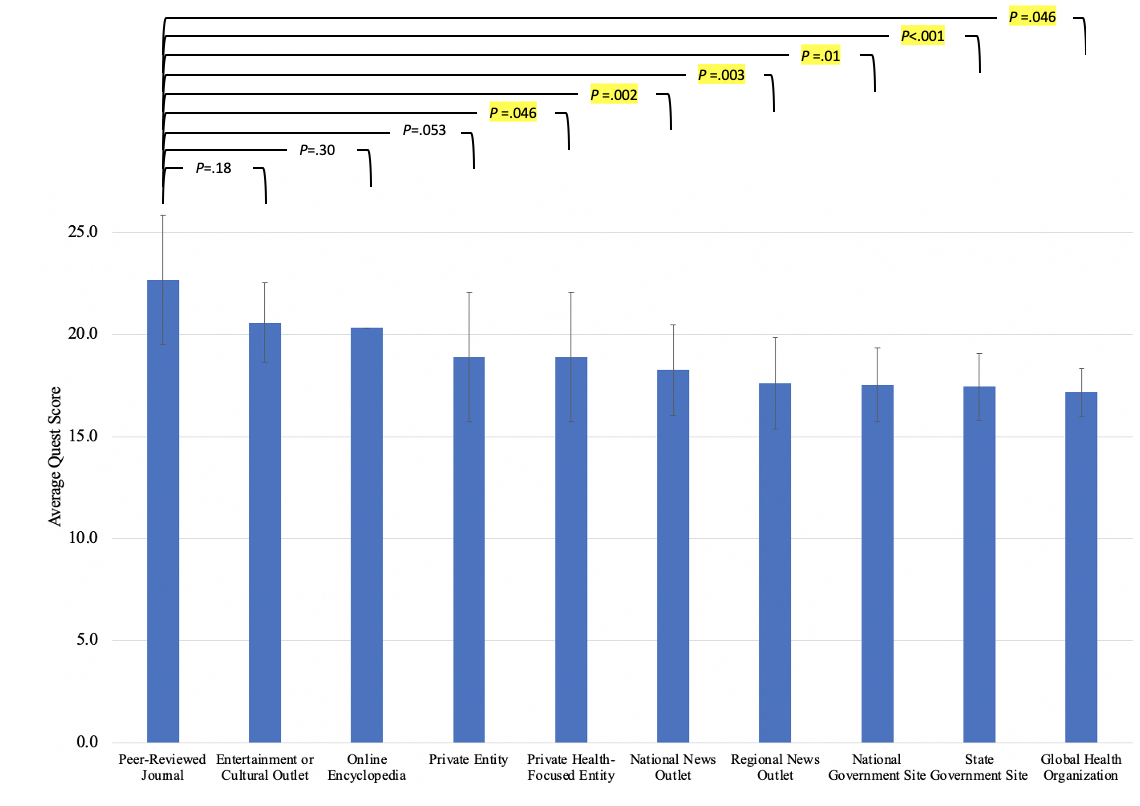
QUEST score by categorization of 195 articles into the article type. Peer-reviewed journal (PRJ; mean 22.7, SD 2.20); entertainment or cultural outlet (mean 20.6, SD 1.95); online encyclopedia (mean 20.3, SD not applicable); private health-focused entity (mean 18.9, SD 3.17); private entity (mean 18.9, SD 3.17); national news outlet (NNO; mean 18.3, SD 2.2); regional news outlet (RNO; mean 17.6, SD 2.24); national government site (NGS; mean 17.5, SD 1.81); state government site (SGS; mean 17.4, SD 1.65); global health organization (GHO; mean 17.2, SD 1.18). PRJ quality score was significantly higher than NNO (*P*=.002), RNO (*P*=.003), NGS (*P*=.001), SGS (*P*<.001), and GHO (*P*=.046) scores. QUEST: Quality Evaluation Scoring Tool.

Categorically, global health organization sites had the lowest average score (mean 17.2, SD 1.2) and least dispersive data set (σ^2^=1.39; Figure 4). Peer-reviewed journals had the most dispersive data set (σ^2^=10.1) and the highest average QUEST score (mean 22.7, SD 3.18), with significantly higher quality averages as compared to national news outlets (mean 18.3, SD 2.20; *P*=.002), regional news outlets (mean 17.6, SD 2.24; *P*=.003), national government sites (mean 17.5, SD 1.81; *P*=.001), state government sites (mean 17.4, SD 1.65, *P*=.002), and global health organizations (mean 17.2, SD 1.18; *P*=.046). In addition, entertainment and cultural outlets (mean 20.6, SD 1.95) also had a significantly higher quality score than regional news outlets (mean 17.6, SD 2.24; *P*=.009) and state government sites (mean 17.4, SD 1.65; *P*=.002).

**Table 1:** Basic descriptive statistics regarding analyzed articles.

Descriptor			Articles, n (%)
**QUEST^a^ scoring fraction**			
	0%-25%	0 (0)	
	25%-50%	22 (11)	
	50%-75%	159 (82)	
	75%-100%	14 (7)	
**Category**			
	National news outlet	70 (36)	
	Private health-focused entity	45 (23)	
	Regional news outlet	29 (15)	
	Private entity	18 (9)	
	State government site	16 (8)	
	National government site	7 (4)	
	Peer-reviewed journal	3 (2)	
	Entertainment or cultural outlet	4 (2)	
	Global health organization	2 (1)	
	Online encyclopedia	1 (<1)	
**Google page**			
	1	47 (24)	
	2	76 (39)	
	3	72 (37)	
**Order on page**			
	0-5	106 (54)	
	6-10	89 (46)	

^a^QUEST: Quality Evaluation Scoring Tool.

**Figure 5:**
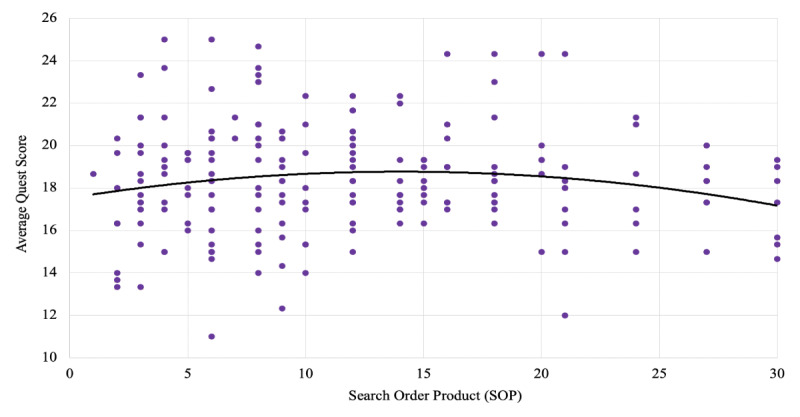
QUEST score stratified by search order product (*R*=–0.16; *P*>.05); there is no hierarchy in score based on the order of search results. QUEST: Quality Evaluation Scoring Tool.

Analysis of the QUEST scores by the search order product showed no significant trends (*R*=–0.16; *P*=.75; Figure 5), discrediting any hierarchy by listing order within the sample set. A significant positive correlation (*R*=0.25; *P*<.001) existed between the number of references (≥1) in an article and the QUEST diagnostic score. Due to QUEST already allocating points in a binary fashion for containing references, only articles with ≥1 reference were considered.

### Readability Analysis

**Figure 6:**
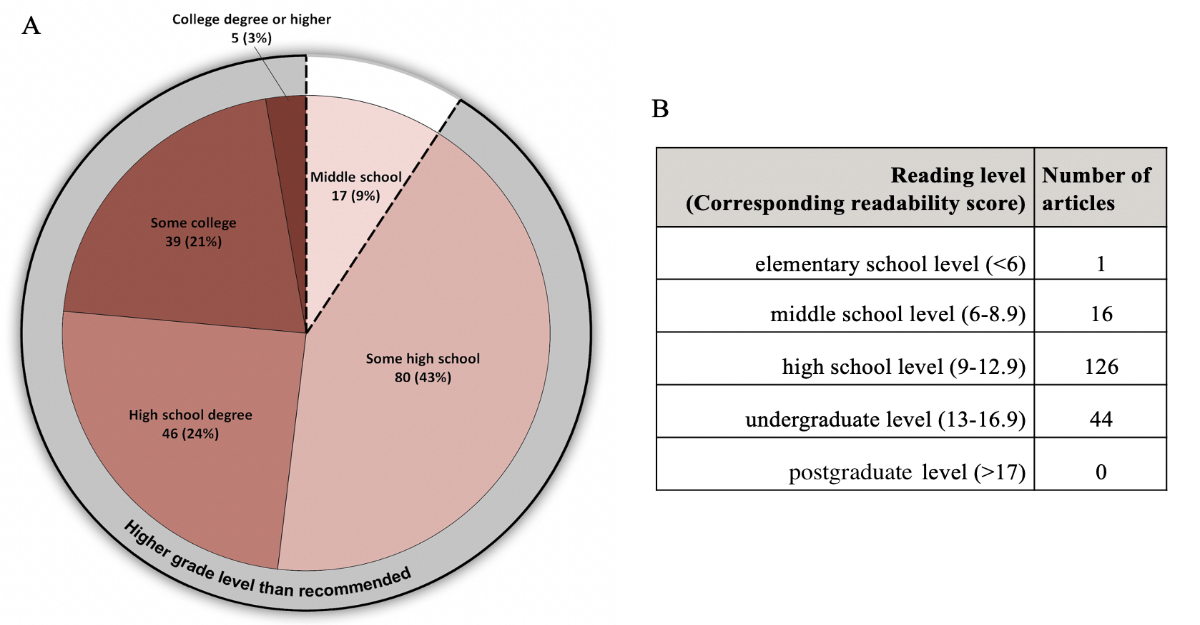
Number of articles (n=187) stratified by educational stages; 187 COVID-19–related articles’ readability score breakdown: middle school, <9th grade; some high school, 9th to 11th grade; high school degree, 12th grade; some college, 13th to 15th grade; college degree or higher, ≥16th grade.

The readability levels for 187 of 195 articles were collected; 8 articles containing document structure unsuitable for analysis were excluded. The average reading level across all 187 articles was 11.7 (SD 1.9), ranging from 5.4 (fifth grade reading level) to 16.9 (undergraduate senior reading level). There was variability among the readability scales, with New Fog Count scoring the overall lowest mean readability (mean 9.3, SD 2.7) and Fry scoring the overall highest mean readability (mean 14, SD 2.9). FRE scored an overall average of 47.2 (SD 11.4), corresponding to difficult and representative of college-level reading. Based on the averages of the nine readability scales for each article, only 3 (1.6%) articles were written at the recommended sixth grade levels [9], with 44 (23.5%) written beyond a high school level (Figure 6).

## Discussion

### Analysis of Results

This study systematically assessed and determined that articles resulting from the most frequently Google-searched health-related COVID-19 terms in the United States were of higher quality and readability than hypothesized. QUEST proved versatile in synthesizing aggregate data regarding different aspects of literature including authorship, attribution, conflict of interest, currency, complementarity, and tone. Despite the high prevalence of misinformation on the internet, analysis of our data set revealed that 89% of articles scored in the upper quartiles, suggesting that online information in the United States regarding COVID-19 was of a higher quality than anticipated.

With the uptick in news dissemination by national media after the onset of the pandemic, national news outlets were unsurprisingly the largest source of our sample set followed by private health-focused entities. Interestingly, there was less output from global organizations such as the World Health Organization and United Nations; these organizations only had 1% of total article output, contradicting their organizational goals of far-reaching public health campaigns and initiatives [24]. This discrepancy in expected and observed output could be due to organizational choices to frequently update a centralized information page as opposed to generating new articles that would show up as separate listings. Additionally, different categorical sources allocate varying levels of resources and personnel to public-facing operations that would result in searchable online information [25].

The lack of significant trends associated with an increasing search order product value implied a qualitatively homogenous sampling of articles by exposure in our analysis, validating the decision to analyze only the first three pages of each term’s Google search (Figure 5). Using the product of search metrics instead of a direct numerical order of results allowed us to place increased weight on articles listed toward the top of later result pages. Among the sample set, the average quality score of the first articles on the first page (mean 18.7, SD 0.0) was not significantly different from the average quality score of the 10th article on the third page (mean 17.1, SD 1.88) of Google search results.

Stratification of the QUEST scores by article categorization revealed source-based qualitative differences, in part due to different data-gathering and publishing processes. Output from peer-reviewed journals had the highest average score (mean 22.7, SD 3.18), likely due to their rigorous scientific vetting process prior to publication [26]. Increased variation in this category (σ^2^=10.1) may be a reflection of the varying requirements set forth by journals and a smaller sample size included in our analysis. Peer-reviewed journals had a significantly increased average QUEST score as compared to national news outlets (mean 18.3, SD 2.20; *P*=.002), regional news outlets (mean 17.6, SD 2.24; *P*=.003), national government sites (mean 17.5, SD 1.81; *P*=.001), state government sites (mean 17.4, SD 1.65; *P*=.002), and global health organizations (mean 17.2, SD 1.18; *P*=.046). Unfortunately, the latter categories were marked by the lowest average information quality, though still mostly in the second (7.5-15) and third (14-22.5) score quartiles. Additionally, entertainment and cultural outlets (mean 20.6, SD 1.95) were characterized by significantly higher quality information compared to regional news outlets and state government sites, perhaps reflecting their tendency to target wider audiences [27].

The majority of articles (170/187, 91%) were written well above American Medical Association–recommended reading levels [21] (Figure 6). Results from the readability analysis substantiated our hypothesis that most COVID-19 articles would be too difficult for the average American to read, in line with the results of prior smaller studies [28]. With the overall FRE score representative of college-level reading and categorically falling under “Difficult,” the ability of these most-searched articles to convey accurate information is automatically diminished. Though not directly contributing to misinformation, mismatched comprehension levels lead to knowledge gaps; this may push the public to turn toward other less reliable modalities to stay informed [29]. Ahmed Siddiqui et al [29] specifically discussed the pervasiveness of nonevidence-based medical advice on social media as a “hidden epidemic” considering its ability to transcend geographic and cultural boundaries. Less readable online sources may indirectly facilitate the spread of misinformation regardless of high article quality. This has been affirmed by studies using different criteria from our own including the DISCERN scoring system [6], JAMA benchmarks [30], and even the HONcode system [31]. Additionally, with massive public health awareness efforts underway to encourage COVID-19 vaccination and safe social practices, accurate online media has become increasingly important as a direct source of information for all demographics [32].

Moving forward, publishing sources may benefit from using resources to optimize communication of health information. The Agency for Healthcare Research and Quality updated the second edition of their Health Literacy Universal Precautions Toolkit (HLUPT) recently in September 2020. The document outlines strategies to enhance overall patient understanding, and even contains a section focused on written communication [33]. In a 2015 study, Brega et al [34] determined that applying the sections of the HLUPT pertaining to written materials led to better readability of revised documents. Subjecting patient-facing articles to rigorous quality and literacy guidelines will aide in improving both publishing standards and consumer understanding, both of which are required to best communicate vital information.

### Conclusion

The COVID-19 pandemic in the United States was accompanied by an influx of online health information. To investigate the need for quality control of this information, we assessed articles resulting from the most-searched health-related terms in the United States using the QUEST rubric and readability software. Despite the high prevalence and transmission of misinformation during the COVID-19 pandemic, the most frequently searched Google articles had good information quality. Still, the majority of these articles were written above the recommended reading level for the public, diminishing their ability to counteract the spread of misinformation.

### Limitations

The limitations of this study include the small sample size, use of only three raters, and lack of individual comparative analysis when determining search keywords. Although GT was used to identify popular keywords, the search volumes of multi-word keywords were not compared against their results without use of quotations. This likely resulted in some bias of listing order because it excluded results only found if only one of the multi-word terms were searched. Furthermore, Google’s newer quality rating guidelines adopted in 2019 have resulted in increased automatic filtering criteria, likely resulting in higher quality and personalized results than would have otherwise been listed [35].

Only the QUEST scale was used to measure article validity, and there are a range of other evaluation tools in the literature that may provide differing or complementary data. Additionally, the Fleiss’s kappa value of our study was 0.0095, indicating a slight agreement between raters when it came to absolute scores. This may have been due to the subjective nature of the QUEST rubric especially in areas such as attribution, tone, and conflict of interest. Even still, author relative rankings of articles correlated to a greater extent than the absolute score values, indicating a shifted but similarly trending rating among raters. Additionally, facets of QUEST, such as authorship and currency, allocate points for characteristics that do not directly correlate with information accuracy, explaining the lower scores of government and global health organizations due to inherent formatting preferences (ie, omitting authors). The QUEST scale does not address every aspect of misinformation, although it does focus on some aspects such as attribution that is seen in Table 1. Further studies on the spread of misinformation, especially against the backdrop of the COVID-19 pandemic, would benefit from examining media outside articles such as radio, social media, and television.

## Appendix

Multimedia Appendix 1 Supplementary data file.

## Abbreviations

FRE: Flesch Reading Ease
GT: Google Trends
HLUPT: Health Literacy Universal Precautions Toolkit
QUEST: Quality Evaluation Scoring Tool
SMOG: Simple Measure of Gobbledygook
